# Dosage Compensation in the Mouse Balances Up-Regulation and Silencing of X-Linked Genes

**DOI:** 10.1371/journal.pbio.0050326

**Published:** 2007-12-11

**Authors:** Hong Lin, Vibhor Gupta, Matthew D VerMilyea, Francesco Falciani, Jeannie T Lee, Laura P O'Neill, Bryan M Turner

**Affiliations:** 1 Chromatin and Gene Expression Group, Institute of Biomedical Research, University of Birmingham Medical School, Birmingham, United Kingdom; 2 BioInformatics and Systems Biology Group, School of Biosciences, University of Birmingham, Birmingham, United Kingdom; 3 Howard Hughes Medical Institute, Harvard Medical School, Boston, Massachusetts, United States of America; 4 Department of Molecular Biology, Massachusetts General Hospital and Department of Genetics, Harvard Medical School, Boston, Massachusetts, United States of America; The Babraham Institute, United Kingdom

## Abstract

Dosage compensation in mammals involves silencing of one X chromosome in XX females and requires expression, in *cis*, of *Xist* RNA. The X to be inactivated is randomly chosen in cells of the inner cell mass (ICM) at the blastocyst stage of development. Embryonic stem (ES) cells derived from the ICM of female mice have two active X chromosomes, one of which is inactivated as the cells differentiate in culture, providing a powerful model system to study the dynamics of X inactivation. Using microarrays to assay expression of X-linked genes in undifferentiated female and male mouse ES cells, we detect global up-regulation of expression (1.4- to 1.6-fold) from the active X chromosomes, relative to autosomes. We show a similar up-regulation in ICM from male blastocysts grown in culture. In male ES cells, up-regulation reaches 2-fold after 2–3 weeks of differentiation, thereby balancing expression between the single X and the diploid autosomes. We show that silencing of X-linked genes in female ES cells occurs on a gene-by-gene basis throughout differentiation, with some genes inactivating early, others late, and some escaping altogether. Surprisingly, by allele-specific analysis in hybrid ES cells, we also identified a subgroup of genes that are silenced in undifferentiated cells. We propose that X-linked genes are silenced in female ES cells by spreading of *Xist* RNA through the X chromosome territory as the cells differentiate, with silencing times for individual genes dependent on their proximity to the *Xist* locus.

## Author Summary

In organisms such as fruit flies and humans, major chromosomal differences exist between the sexes: females have two large, gene-rich X chromosomes, and males have one X and one small, gene-poor Y. Various strategies have evolved to balance X-linked gene expression between the single X and the autosomes, and between the sexes (a phenomenon called dosage compensation). In Drosophila melanogaster, expression from the male X is up-regulated approximately 2-fold, thereby balancing both X-to-autosome and female-to-male expression. In contrast, mammals silence one of the two female Xs in a process requiring the untranslated RNA product of the *Xist* gene. This balances female-to-male expression but leaves both sexes with only one functional X chromosome. Using mouse embryonic stem cells and microarray expression analysis, we found that dosage compensation in mice is more complex than previously thought, with X-linked genes up-regulated in both male and female cells so as to balance X-to-autosome expression. As differentiation proceeds, female cells show progressive loss of expression from one of the two initially active Xs. Surprisingly, silencing occurs on a gene-by-gene basis over 2–3 week of differentiation; some genes escape altogether, whereas a subgroup of genes, often adjacent to the *Xist* locus, is silenced even in undifferentiated cells. We propose that female X-linked genes are silenced by progressive spreading of *Xist* RNA through the X chromosome territory as differentiation proceeds.

## Abstract 3

In mouse embryonic stem cells, X:autosome expression balance is achieved by up-regulating X-linked genes in both sexes and gene-by-gene silencing on one female X chromosome.

## Introduction

In many higher eukaryotes, sex determination mechanisms have evolved in a way that has generated chromosomal differences between the sexes. In eutherian and marsupial mammals and the fruit fly *Drosophila*, females have two copies of a gene-rich X chromosome, whereas males have one X and one smaller, gene-poor Y. Because monosomy for even the smallest autosome is lethal in mammals, mechanisms have presumably evolved to allow males to tolerate monosomy of the X, as well as to correct a potential imbalance between the sexes in expression levels of several hundred X-linked genes [1,2]. In *Drosophila*, the situation has been resolved by an overall up-regulation of genes on the single male X, a dosage compensation mechanism that equalises expression both between X and autosomes and between the sexes [3,4]. In mammals, expression in males and females has been balanced by X inactivation, a process by which most genes on one of the two female Xs are silenced early in development [5–7]. However, X inactivation alone exacerbates the X:autosome imbalance, leaving both sexes functionally monosomic for X-linked genes. This problem was highlighted many years ago, and a balancing, 2-fold up-regulation of genes from the single, active X was proposed as a possible solution [2,8]. However, proof of this has been difficult to achieve. Studies of the expression of the *Ccl4* gene in hybrid mice provided a clue that this might occur [9], but the first indication that genes on the active X are globally up-regulated has come only recently through the analyses of microarray data from a variety of publicly available sources. Comparisons of the mean, overall expression levels of X-linked and autosomal genes in various cell and tissue types, usually from mixtures of male and female, gives an X:autosome expression ratio of approximately 1 [10–12]. Given that both XY male and XX female cells have only a single, transcriptionally active X, and two copies of each autosome, without up-regulation of X-linked genes the mean ratio should be closer to 0.5. The results therefore provide evidence, albeit circumstantial, for a balancing up-regulation of expression from the active X.

We used microarray expression analysis to give a global picture of X-linked gene expression in differentiating mouse embryonic stem (ES) cells, a model system that allows the dynamics of dosage compensation processes to be analyzed [13]. We show that up-regulation of X-linked genes is in place in undifferentiated male and female ES cells but is incomplete, with equalization of X-linked and autosomal transcript levels requiring 2–3 wk of differentiation. Evidence for a similar up-regulation was found in inner cell mass (ICM) cells isolated from male and female blastocysts. In female ES cells, continuing up-regulation through differentiation is counterbalanced by silencing of genes on the second, randomly chosen X. We show that silencing of X-linked genes occurs on a gene-by-gene basis throughout differentiation, and we present evidence that silencing is mediated by the progressive spreading of *Xist* RNA through the X chromosome territory.

## Results

### Expression of X-Linked Genes Is Up-Regulated in Both Female and Male ES Cells

**Figure 1:**
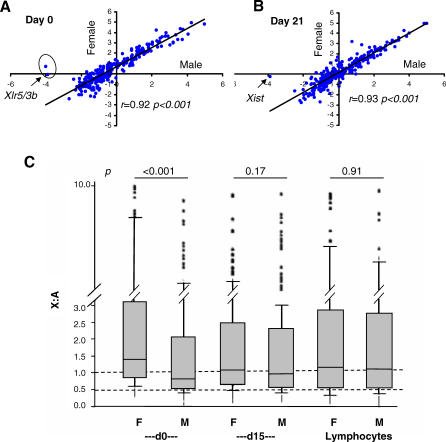
Expression of X-Linked Genes Is Consistently Higher in Female than in Male ES Cells Prior to Differentiation (A and B) Correlation in expression levels of X-linked genes between female (*y*-axis) and male (*x*-axis) ES cells. The value for each of the 249 clones (180 genes) shown is expressed relative to the expression of autosomal genes (X:A ratio, log_2_ scale). Two genes, *Xlr3b* and *Xlr5*, showed female-specific expression prior to differentiation (panel A). *Xist* is weakly expressed overall, but relatively more strongly expressed in differentiated female ES cells than in males (panel B). Pearson product moment correlation coefficients (*r* values) are shown, along with estimates of the probability of chance correlation (*p*) corrected for false discovery rate (FDR). (C) Box plots (linear scale) showing the distribution of expression of X-linked genes in female and male ES cells before (day 0) and after (day 15) differentiation and in lymphocytes. The box encompasses the 25th–75th percentile and the upper and lower lines represent the 10th–90th percentile. Outlying values are shown as individual dots. Data are from at least two biological replicates.

Expression of X-linked genes in female and male ES cells, relative to autosomal genes (X:A ratio), was assayed by co-hybridisation of labelled cDNAs to NIA15K mouse cDNA microarrays [14]. Expression levels of 180 X-linked genes (see Text S1 for filtering criteria) were distributed over a ∼200-fold range, with a close correlation between expression in female and male cells (Figure 1A and 1B). Only two genes showed clear sex-specific expression in undifferentiated ES cells, namely *Xlr3b* and *Xlr5*, both of which showed minimal expression in the CCE/R male line. Consistent over-expression of *Xlr5c* and *Xlr5d* in female ES cells relative to males has recently been reported by others [15]. On differentiation, the expression of both these genes increased progressively in male CCE/R cells and decreased in female PGK12.1 cells, such that by day 21, expression was at comparable levels in both cell types (Figure S1A). Interestingly, *Xlr3b* and *Xlr5* are part of a cluster, some of whose members show tissue-specific imprinting [16]. For undifferentiated cells (Figure 1A), the regression line intercepts the *y*-axis (female) at a positive value (log_2_ = 0.69, linear 1.61), showing that overall expression of X-linked genes is 1.6-fold higher in undifferentiated female ES cells, with two active X chromosomes, than in male ES cells, with only one. In differentiated cells (Figure 1B), the intercept was close to 0 (log_2_ = 0.14, linear 1.10), indicating that overall expression of X-linked genes was very similar in differentiated male and female cells.

The distributions of expression levels of X-linked genes in male and female ES cells are shown as box plots in Figure 1C. In both males and females, expression levels are skewed towards higher expression levels (Figure S2). A similar skewing was seen when the expression of genes on individual autosomes was analysed in the same way (Figure S2). To accommodate this, we have used median rather than mean values for comparison (Figure 1C, horizontal line in each box). The median X:A ratio in undifferentiated (day 0) male ES cells is 0.81 (Figure 1C). This is significantly above the median value of 0.5 that would be expected if X-linked genes were expressed equally to autosomal genes (*p* = 3.2 × 10^−32^, *n* = 249) and demonstrates an overall 1.6-fold up-regulation of expression on the single X. Microarray expression data for three additional male ES cell lines has recently been made available (BL6.9, 129.3, and ES-D3-GL) [15], and we have used this to calculate X:A expression ratios, as above. Values ranged from 0.90 to 1.03, suggesting that an approximately 2-fold up-regulation of X-linked genes is a general property of male ES cell lines.

In XX female cells, equal expression of X-linked and autosomal genes would give an X:A ratio of about 1.0, whereas the measured ratio is 1.39, which is significantly higher (Figure 1C, *p* = 1.1 × 10^−6^, *n* = 249). A very similar X:A expression ratio (1.37) was given by cDNA from the hybrid, female ES cell line 3F1 (see below). Thus, X-linked genes in female ES cells are also up-regulated. After 15 d of differentiation, X:A ratios in both male and female cells were close to 1, which is comparable to the situation in adult cells (Figure 1C).

### Expression of X-Linked Genes in the ICM

**Figure 2:**
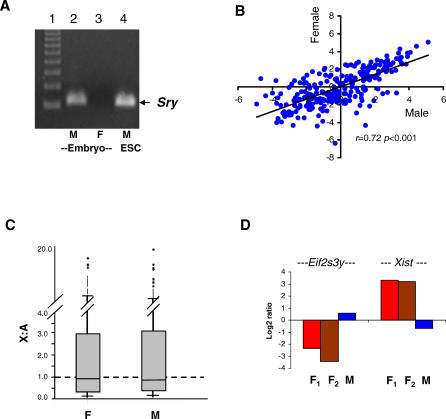
Expression of X-Linked Genes in Male and Female ICM (A) Gel showing presence or absence of PCR products derived from the *Sry* gene in trophectodermal material from single blastocysts used for ICM cDNA preparation. Lane 1, 123 bp size markers; lane 2, male embryo; lane 3, female embryo; lane 4 male ES cell line CCE/R. (B) Correlation in expression levels of X-linked genes between female (*y*-axis) and male (*x*-axis) ICMs. The value for each gene is expressed relative to the expression of autosomal genes (X:A ratio, log_2_ scale). Pearson product moment correlation coefficient (*r* value) and FDR-corrected probability of chance correlation (*p*) are shown. (C) Box plot showing expression of X-linked genes (X:A ratio) in male and female ICMs. The box encompasses the 25th–75th percentile, and the upper and lower lines represent the 10th–90th percentile. Outlying values are shown as individual dots. Female values are from two biological replicates and male values from two technical replicates. (D) Expression of the Y-encoded, male-specific antigen Eif2s3y and Xist RNA, relative to autosomal genes (log_2_ ratio), in ICMs from female and male embryos.

To test whether the up-regulation of X-linked genes detected in ES cells also occurs in the cells of the blastocyst from which ES cells are derived, RNA was prepared from single ICMs from cultured male and female embryos (distinguished by presence or absence of the *Sry* gene, Figure 2A), amplified and used to prepare cDNA for labelling of NIA15K arrays. As with expression in ES cells, the expression of X-linked genes in female and male embryos was closely correlated (Figure 2B). The median X:A ratio in male ICMs was 0.86 (Figure 2C), very similar to the value of 0.81 obtained for male ES cells (Figure 1C) and indicative of up-regulation of X-linked gene expression. In female ICMs from the same batch of embryos, the X:A ratio was 0.89 (Figure 2C). This is consistent with up-regulation of genes on Xa in females only if one of the two Xs is inactivated in all or most of the ICM cells used in this experiment. This is certainly possible, because levels of *Xist* RNA were particularly high in female ICMs (around 8-fold higher than the median expression of autosomal genes) and significantly higher than the *Xist* RNA levels in male ICM (Figure 2D) and differentiated female ES cells (Figure 1B). Whether this represents persistence of the imprinted paternal X inactivation present from early in development, or random X inactivation, or a combination of the two [17], remains to be determined.

### Changes in Expression of X-Linked Genes Proceed throughout ES Cell Differentiation

**Figure 3:**
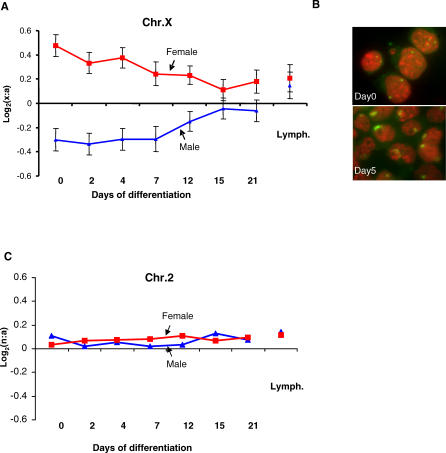
Dynamics of Change in X-Linked Gene and Autosomal Gene Expression during ES Cell Differentiation (A) Expression of X-linked genes (X:A ratio, log_2_ scale) in female (red) and male (blue) ES cells at different stages of differentiation from day 0 (undifferentiated) to day 21. Each data point (± SE) is the averaged median value from 5 (day 21), 4 (day 0), 3 (day 12), or 2 (days 2, 4, 7, 15) biological replicates. (See Text S1 for details). (B) RNA FISH showing distribution of *Xist* RNA (green) in undifferentiated (day 0, upper panel) and differentiating (day 5, lower panel) female ES cells, counterstained with DAPI (red). The prominent, widely spread *Xist* signal was detected in ∼60% of day 5 cells. (C) Expression of genes on Chromosome 2 (n:A ratio, log_2_ scale) in female (red) and male (blue) ES cells at different stages of differentiation. Replicates as in (A).

The dynamics of differentiation-related changes in X-linked gene expression were determined by analysing cDNAs from male and female cells at various times of differentiation between 0 and 21 d. The X:A expression ratio in female cells showed a gradual and progressive decrease until day 15, whereas in male cells, there was little change until day 7, after which there was a progressive increase (Figure 3A). The gradual changes in X-linked gene expression contrast with the relatively early change in distribution of *Xist* silencing RNA detected by RNA–fluorescence in situ hybridisation (FISH) (Figure 3B) and loss of expression of the pluripotency markers *Nanog*, *Pou5f1/Oct4*, and *Zfp42*/*Rex1* (Figure S1B). *Xist* RNA levels increased through differentiation from day 2 onwards (Figure S1C).

The observed increase in expression of X-linked genes in differentiating male cells and the decrease in female cells are unique properties of X-linked genes. Expression of genes on each of the 19 mouse autosomes, relative to all genes (designated the n:A ratio) showed no such changes. The n:A ratio varied from one chromosome to another over only a narrow range and did not change with differentiation or differ between females and males to the same extent as did the X:A expression ratio (Chromosome 2 is shown as an example in Figure 3C and all 19 autosomes are shown in Figure S3).

**Figure 4:**
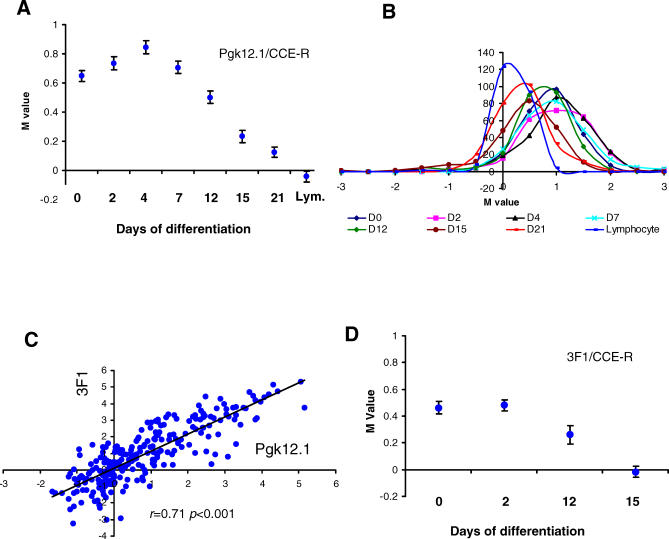
A Fall in X-Linked Gene Expression in Female ES Cells Is Detected Late in Differentiation (A) M values (female:male expression ratio, log_2_ scale, ±SE) for X-linked genes in PGK12.1 female ES cells at different times of differentiation (days 0–21). The fall in M value is attributable to the progressive inactivation of genes on one of the two female Xs. Biological replicates for PGK12.1 cells are as listed in Figure 3A. (B) Distribution of M values in PGK12.1 ES cells at different days of differentiation (day 0, day 2, etc., as indicated) and adult lymphocytes. All distributions are normal with a progressively decreasing median. (C) Correlation in expression levels of X-linked genes between 3F1 (*y*-axis) and PGK12.1 (*x*-axis) ES cell lines. The value for each X-linked gene is expressed relative to the expression of autosomal genes (X:A ratio, log_2_ scale). Pearson product moment correlation coefficient (*r* value) and FDR-corrected probability of chance correlation (*p*) are shown. (D) M values (female:male expression ratio, log_2_ scale, ±SE) for X-linked genes in 3F1 female ES cells at different times of differentiation (days 0, 2, 12, and 15).

The expression of X-linked genes in differentiating female ES cells can potentially be influenced by the following three parallel processes: (i) silencing through X inactivation, (ii) up-regulation through dosage compensation on the active X, and (iii) differentiation-related expression changes that are unconnected to the dosage compensation process. Given the close correlation between expression levels of X-linked genes in male and female cells throughout differentiation (Figure 1A and 1 B), the latter two processes are likely to occur to a similar extent in both male and female cells. This being the case, changes in the female:male expression ratio of X-linked genes should reflect progression of the X inactivation process alone. With this in mind, we co-hybridised red/green labelled cDNAs from female and male cells, at the same stage of differentiation, to the same slide, and we calculated red:green or green:red ratios as a log_2_ “M value”, as described [18,19]. In undifferentiated, cells the M value is around 0.65 (Figure 4A), corresponding to a linear female:male expression ratio of about 1.6. M values derived from undifferentiated cells and cells at later stages of differentiation were all normally distributed (Figure 4B). There was no detectable fall in M value for the first 7 d of differentiation, with a small increase at days 2–4. Thereafter, there was a progressive decrease, culminating in an M value close to 0, which reflects equal expression of X-linked genes in female and male cells by day 21 (Figure 4A and 4B). It seems that a net loss of expression of female X-linked genes occurs later than previously concluded on the basis of single-gene analyses [13,20].

To determine the consistency of these findings between ES cell lines, we assayed X-linked gene expression in the hybrid (*m. mus domesticus* × *m. mus castaneous*) ES cell line 3F1. There is a close correlation in expression of X-linked genes between these two very different lines (Figure 4C). Further, co-hybridisation of 3F1 and male (CCE/R) cDNAs from the same stages of differentiation to the same slides showed a relatively late decrease in the expression of female X-linked genes relative to male, with no detectable decrease in female:male expression ratio (M value) after 2 d of differentiation, and complete equalisation only after 15 d (Figure 4D).

By studying the change in M value with differentiation time for individual genes, it became clear that some genes consistently showed a relatively early loss of activity in female cells, while others inactivated later, or not at all. Differences in silencing times were confirmed by real-time quantitative (RTQ)-PCR assays, in which expression levels in differentiating embryoid bodies were expressed relative to levels at day 0 (examples are presented in Figure S4). As a first test of whether genes that inactivated relatively early in one ES cell line also inactivated early in others, we co-hybridised cDNA from undifferentiated cells and cells differentiated for 7 d to the same slide and calculated the day 7:day 0 expression ratio as an M value. Genes showing reduced expression in PGK12.1 cells after 7 d of differentiation tended also to show reduced expression in 3F1 cells, with a good overall correlation between the two cell lines in the change in expression of X-linked genes after 7 d of differentiation (*p* < 0.001, Figure S5).

**Figure 5:**
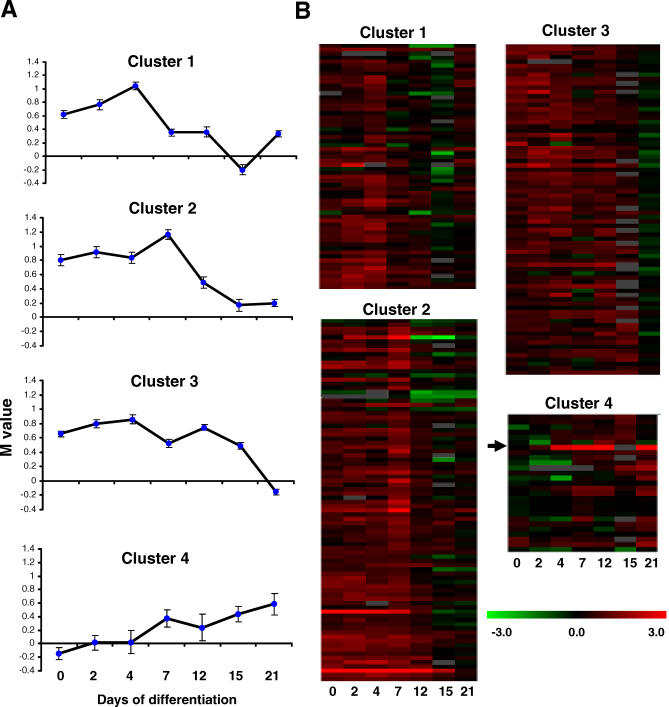
Genes Are Silenced at Widely Differing Times during Differentiation of Female ES Cells (A) X-linked genes on the array were separated into four clusters on the basis of their patterns of change in female:male expression ratio (M values) during ES cell differentiation. Median values (± SE) for each cluster at each time point are shown. Changes in M value are attributable to the progressive inactivation of genes on one of the two female Xs. (B) Heat maps for each gene, with female:male expression ratio colour-coded from green (lowest) to red (highest) as shown on the coloured bar (lower right). *Xist* (cluster 4, arrowed) shows relatively strong expression in female cells from early in differentiation.

We subjected the PGK12.1 M value dataset (Figure 4A) to cluster analysis using the TIGR programme [21]. The programme grouped expression data from 252 X-linked clones into selected numbers of clusters, based on the manner in which expression (M value) changed during differentiation. Figure 5A shows the results of an analysis in which the data were resolved into four clusters, each shown as a graph plotting the median value at each stage of differentiation tested. Figure 5B shows the corresponding heat maps. Clusters 1, 2, and 3 showed similar patterns of change, starting at an M value of 0.6–0.8 at day 0 (corresponding to a linear F:M ratio of 1.54–1.75) falling to around 0 by day 21, but differing in the stage at which M values first fell significantly, i.e., day 4–7 for cluster 1 (46 genes), day 7–12 for cluster 2 (74 genes), and day 12–21 for cluster 3 (64 genes). In contrast, the 21 genes in the fourth cluster behaved differently, with M values close to 0 in undifferentiated cells (median = −0.14) and generally increasing on differentiation (Figure 5A). We note that irrespective of how many clusters the programme was asked to resolve, there was always one that showed essentially the same pattern as that of cluster 4 and that stood out from the rest. The genes in cluster 4 are listed in Table S1. They include *Xist*, a gene known to show increased expression in female cells as they differentiate [22] (Figure S1C). Ontology analysis using Fatigo+ [23] showed that none of these four gene clusters was significantly enriched in genes associated with specific functional categories or cell lineages (unpublished results).

### Some X-Linked Genes Show Mono-Allelic Expression in Undifferentiated Female ES Cells

One explanation (among several) for the finding that some genes are equally expressed in XX female and XY male ES cells is that they are expressed from only one allele in female cells. This could result from the failure to reactivate the paternal allele which, for most X-linked genes, is selectively silenced in the preblastocyst embryo but reactivated in the inner cell mass during blastocyst maturation [17,24]. Alternatively, it could reflect initiation of (random) X inactivation before the onset of ES cell differentiation. To explore these possibilities, we assayed the expression of paternal and maternal alleles using the hybrid ES cell line 3F1, derived from a *m.mus domesticus (129/sv)* × *m.mus castaneous* hybrid backcrossed to *129* [25]. The *129* X chromosome is maternally derived, whereas the *castaneous* X chromosome is paternal [26]. In 3F1 cells, the *129* X chromosome carries a loss-of-function *Tsix* mutation, such that when 3F1 cells differentiate, *Xist* is always up-regulated on the *129* X chromosome, which is therefore always inactivated [25].

**Figure 6:**
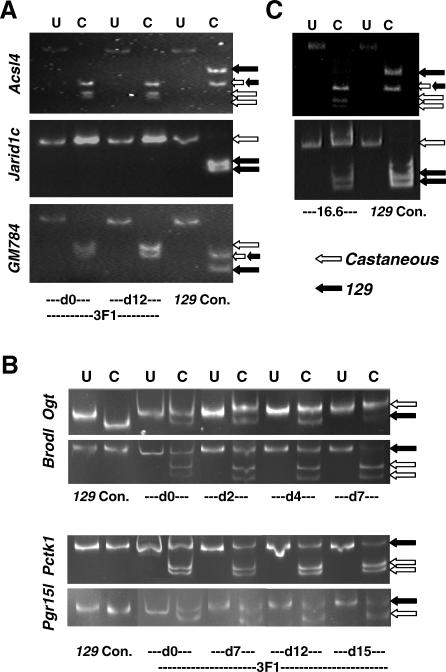
Allele-Specific Analysis of X-Linked Gene Expression during Differentiation of Hybrid (*129* × *castaneous*) Female ES Cells (A) Electrophoretic separation of cDNA restriction fragments from the three cluster 4 genes *Acsl4*, *Jarid1c*, and *GM784* reveals allele-specific expression. PCR fragments that are specific for each gene were prepared from cDNAs from undifferentiated (day 0) or differentiated (day 12) 3F1 hybrid ES cells. Fragments were uncut (U) or cut (C) with restriction enzymes, so as to reveal SNPs that distinguish *m.m.domesticus* (*129*, black arrows) and *m.m.castaneous* (white arrows) alleles. Small double arrows show where bands of the same size are generated from *129* and *castaneous*. For all three genes, the expected *129* product is missing from both undifferentiated (day 0) and differentiated (day 12) cells, showing that expression is exclusively from the castaneous allele. cDNA from the male CCE/R ES cell line provided the *129* control. Details of primers, restriction enzymes, and expected fragment sizes are given in Table S5. (B) Electrophoretic separation of cDNA restriction fragments from four X-linked genes *Ogt*, *Brodl*, *Pctk1*, and *Pgr15l* to reveal allele-specific expression in 3F1 hybrid female ES cells at different days of differentiation (days 0–15 as indicated); labelling as for (A). (C) In undifferentiated 16.6 hybrid female ES cells, cluster 4 gene *Acsl4* (upper gel) shows monoallelic expression (*castaneous* allele only) while *Jarid1c* (lower gel) is expressed from both alleles; labelling as for (A).

We identified single nucleotide polymorphisms (SNPs) in three cluster 4 genes (*Jarid1c*, *Gm784*, and *Acsl4*) that distinguished the *129* and *castaneous* alleles and that could be selectively restriction digested so as to generate cDNAs that are distinguishable electrophoretically . Remarkably, for all three genes, expression in undifferentiated 3F1 cells was exclusively from the *castaneous* allele (Figure 6A). We were able to test three other genes that showed a female:male expression ratio of close to 1 at day 0, but which the TIGR programme had not placed in cluster 4. One of these, *Phka2*, showed expression exclusively from the *castaneous* allele in undifferentiated 3F1 cells; a second, *Ogt*, showed expression that was strongly skewed towards the *castaneous* allele; whereas the third, *Brodl*, showed bi-allelic expression (Figure 6B). Two genes whose female:male expression ratios in undifferentiated cells showed the expected female bias by microarray analysis (*Pctk1* and *Zfp185*) showed clear biallelic expression, as did a third gene (*Pgr15l*), for which a suitable SNP was available but which was not present on the NIA15K array (Figure 6B, supplementary Figure S6). The conclusion from these results is that a subpopulation of X-linked genes in female ES cells is mono-allelically expressed before differentiation and that in 3F1 cells, where inactivation is 100% skewed towards the maternal X, it is always the paternal (*castaneous*) X that is expressed. Thus, mono-allelic expression is not due to failure to reactivate the paternal allele from its preblastocyst silent state, but instead is due to the onset of “random” X inactivation before differentiation.

For all genes tested that showed biallelic expression in undifferentiated cells (*Brodl*, *Zfp185*, *Ogt*, *Pctk1*, and *Pgr151*), allele-specific analysis confirmed the microarray data, showing that inactivation of X-linked genes occurs over a wide range of differentiation times and that individual genes have characteristic times of inactivation (Figure 6B and Figure S6).

### Proximity to the X inactivation centre (Xic) Correlates with Mono-Allelic Expression in Undifferentiated Female ES Cells

In searching for possible reasons for the gene-to-gene differences in inactivation rate, we asked whether position on the X chromosome, and specifically proximity to *Xist* and the Xic, was of any relevance. To do this, we tabulated the distribution of genes in each cluster across seven X chromosome regions of similar gene content (Table S2). Genes in clusters 1–3 are distributed across the X chromosome with no clear enrichment or depletion in any single region, nor any clear differences between clusters. In contrast, cluster 4 showed a significant enrichment in the region (85–108Mb) that contains the Xic (8 of 21 genes, *p* = 0.038, Fisher's exact test). Six of these eight genes are within 6 Mb (94.8∼100.5 Mb) of the Xic (Tables S1 and S2).

The tendency of genes silenced before ES cell differentiation to be located adjacent to the Xic suggests that *Xist* RNA plays a role in their silencing, even before its increased expression early in differentiation. To test this, we took advantage of the finding that *Xist* transcript levels in undifferentiated 3F1 cells (carrying a mutation of the *Tsix* gene) are about 3-fold higher than in the 16.6 hybrid line, from which 3F1 was derived [25] and which has a functional *Tsix* gene [22]. In 16.6 cells, inactivation is 80% skewed towards the *129* X chromosome as a result of differences in strength of the *Xce* alleles in the parental strains [27,28]. In undifferentiated 16.6 cells, two of the four genes that are mono-allelically expressed in 3F1 cells (*Gm784*, *Acsl4*) were also expressed exclusively from the *castaneous* allele. However, the two that were most distant from the Xic (*Phka2*, *Jarid1c*) were bi-allelically expressed (examples shown in Figure 6C), consistent with the possibility that silencing reflects local spreading of *Xist* RNA.

If silencing of X-linked genes reflects the progressive spreading of *Xist* RNA through the X chromosome territory, then one would predict that genes that are close together on the chromosome should be silenced at similar times. To test this, we prepared a list of X-linked gene pairs separated by progressively increasing distances, and we asked whether members of each pair were found in the same cluster (i.e., any one of clusters 1–4, Figure 4) more often than expected by chance. For this analysis, the four clusters are taken as broad indicators of inactivation timing. We find that gene pairs separated by up to 40 kb (Table S3) are in the same cluster significantly more often than predicted by chance (*p* < 0.05, statistical procedures used are outlined in Text S1 and Figure S7). The five closest gene pairs, from 0 to 2.8 kb apart, were always present in the same cluster (*p* < 0.05, Table S3).

## Discussion

The results presented here use microarray expression analysis to show that dosage compensation in mouse ES cells involves up-regulation of X-linked genes in both males and females, together with the progressive, differentiation-dependent silencing of genes on one of the two female X chromosomes. We find that up-regulation is present prior to ES cell differentiation and is present also in male (and most likely female) ICMs. Up-regulation of X-linked gene expression in male and female cells is consistent with the overall ∼1:1 expression ratio of X-linked and autosomal genes calculated from analysis of publicly available microarray expression data for a wide range of mouse and human cell types [11].

### Progressive Silencing of X-Linked Genes in Differentiating Female ES Cells

Increasing the level of *Xist* RNA transcripts early in differentiation of female ES cells, is a key event in silencing, in *cis*, of X-linked genes [6,22]. The more extensive *Xist* signal detected by RNA-FISH, following up-regulation, has been thought to represent “coating” of the X chromosome, an event that triggers gene silencing. However, the data presented show that global silencing of X-linked genes in differentiating ES cells is not contiguous with the onset of *Xist* up-regulation, but is put in place progressively over several weeks of differentiation. Indeed, we find no general relationship between transcriptional silencing and any one of the chromosome-wide changes that first become apparent on Xi at specific stages of ES cell differentiation, including *Xist* coating and H3 lysine 9 methylation (days 1–2), histone deacetylation (days 5–7), and incorporation of the histone variant macroH2A (days 8–12) [20,29–31].

The slow, progressive fall in X-linked transcripts is not easily attributable to experimental or technical factors. Falls in transcript levels inevitably lag behind transcriptional silencing and will show a spread of values that reflects how promptly individual cells begin to differentiate, but such effects cannot account for the consistent variation from one gene to another in the stage at which transcript levels fall. Nor can differences in RNA turnover or stability account for the gene-to-gene variation in silencing time. An unstable transcript will vanish as soon as transcription stops, while a completely stable transcript will be diluted 2-fold at each cell division (i.e., every 16–18 h). Even these extreme stability differences cannot explain variations in inactivation time spread over 3 wk of differentiation. Strain-related differences in global gene expression patterns in different ES cell lines have recently been carefully documented [15], but we find no evidence that differences between ES cell lines, or the mouse strains from which they were derived, are fundamentally influencing our results. Levels of expression of the X-linked genes on the NIA15K array were very closely correlated between CCE/R (*129/sv*), PGK12.1 (*PGK* × *129/OLA*), and 3F1 (*129* × *m.mus castaneous*) ES cells, whereas both overall up-regulation of X-linked gene expression prior to differentiation and the slow decrease in X-linked gene expression during differentiation were similar in PGK12.1 and 3F1 cells.

Our observation that some genes (e.g., *Jarid1c* and *Acsl4*) are silenced on the chosen X in undifferentiated ES cells shows that up-regulation of *Xist* expression is not essential for silencing. However, the tendency of these early-silencing genes to lie close to the *Xist* locus suggests that *Xist* RNA may still be involved, possibly through local spreading, the extent of which is limited by the low level of *Xist* expression in undifferentiated cells [22]. This possibility is consistent with the finding that whereas four genes were shown by allele-specific analysis, to be mono-allelically expressed in undifferentiated 3F1 cells, only two of these show mono-allelic expression in 16.6 cells, in which *Xist* transcript levels are 3-fold lower [22]. The two genes that escape silencing in 16.6 cells (*Phka2* and *Jarid1c*) lie furthest from *Xist*, a finding generally consistent with a local spreading model. These results also raise the possibility that *Jarid1c* silencing is particularly sensitive to *Xist* expression levels, which might help explain reports that this gene (previously known as *Smcx*) escapes inactivation to varying degrees depending on stage of differentiation and cell or tissue type [32–34].

Recent data suggest that up-regulation of *Xist* RNA early in ES cell differentiation leads to the formation of a distinct *Xist* domain within the X chromosome territory, into which genes are placed as they are silenced [20,35]. The results presented here are consistent with an *Xist* domain, but we suggest that the domain expands through the X chromosome territory as differentiation proceeds, and that genes are silenced as they come into contact with the spreading *Xist* RNA. The progressive increase in *Xist* transcript levels during differentiation of female, but not male, ES cells (Figure S1C) is consistent with an expanding *Xist* domain. The stage at which any gene is silenced will therefore depend on its position within the X chromosome territory relative to the *Xist* locus. These positions will depend on how the X chromosome is folded within its territory, and for all genes except those most proximal to *Xist*, the folding need bear little relationship to the gene's (linear) position on the chromosome, which we find to be the case (Table S2). We do find, as predicted by the spreading model, that genes that lie close together on the chromosome (within about 40 kb) tend to be silenced at about the same time, as measured by cluster analysis (Table S3). None of this evidence is inconsistent with the possibility that some genes at least may be actively drawn into the *Xist* domain [20]; it is possible that *Xist* spreading and gene repositioning occur in parallel.

In the model we propose, the pattern of gene silencing through differentiation is critically dependent on the configuration of the X chromosome territory, specifically the positioning of the *Xist* locus and of other loci relative to it. Differential reconfiguration of X chromosome territories in female cells prior to the onset of X inactivation, or even changes in their intranuclear location, may be a crucial initial step in the X inactivation process [22,36]. The fact that XY male ES cells express low levels of *Xist* RNA prior to differentiation, but do not inactivate genes proximal to *Xist* (e.g., *Gm784*, *Acsl4*, Figure 6), indicates that *Xist* RNA is not the sole determinant of inactivation. Perhaps configuration of the X chromosome territory or chromatin conformation in undifferentiated male ES cells is such as to preclude contact between *Xist* RNA and critical X-linked loci. Our previous observation that X-linked genes in female ES cells carry levels of histone modifications associated with transcriptional activity that are higher than those in males [37] raises the intriguing possibility that chromatin modifications might help determine susceptibility to *Xist* silencing. In this respect, it is interesting that the dosage compensation complex in D. melanogaster, which includes *roX* RNAs, preferentially targets transcriptionally active genes, possibly through their distinctive histone modifications [3,4,38].

### Evolutionary Considerations

Dosage compensation is a rapidly evolving process, and the mechanisms by which it is accomplished vary from one organism to another [1,10,38]. It is interesting to ask whether evolution is driven predominantly by a need to equalise overall X-linked and autosomal expression levels, or whether transcript levels of key individual genes exert the major selection pressure. Recent studies on expression of Z-linked genes in birds, in which females are heterogametic (ZW) and males homogametic (ZZ), throw some interesting light on this [39]. For a representative group of genes in various tissues in two species (zebra finch and chicken), the expression of Z-linked genes was consistently and significantly higher in ZZ males—where the Z:autosome expression ratio was around 1—than in ZW females—where the Z:autosome ratio ranged from 0.7 to 0.9 depending on the tissue. These findings indicate that dosage compensation is incomplete in birds, and that higher eukaryotes can tolerate significant overall differences in gene expression between the sexes and between X-linked and autosomal genes.

It now seems that the three model organisms commonly used to study dosage compensation: fruit fly (D. melanogaster), mouse (Mus musculus), and the nematode worm Caenorhabditis elegans have all adopted up-regulation of X-linked gene expression in XY (or XO) males as a means of balancing X:autosome expression levels [1,38]. In mouse and C. elegans [40,41] there is also an overall suppression of X-linked transcription in XX females/ hermaphrodites. The extra complexity of the mammalian, and worm, mechanisms is likely to reflect their evolutionary histories. It is generally accepted that the gene-poor Y chromosome is the evolutionary result of progressive degeneration of one of two originally homologous chromosomes, one of which (the proto-Y) carried a sex-determining allele [42,43]. Restricted crossing-over at and around the sex-determining locus, which is necessary to prevent the formation of intersex states, allows the progressive spread of mutations and the loss of functional genes along the proto-Y by reducing the selection pressure to which they are subjected [8]. For many mutated genes, selection pressure will favour up-regulation of the remaining functional allele to restore the original transcript levels. The magnitude of this selection pressure will depend on the sensitivity of the gene product's function to transcript level. If in mammals (and C. elegans), unlike *Drosophila*, the newly evolving up-regulation mechanism were expressed from the beginning in both males and females, then a female-specific silencing mechanism would need to evolve in parallel to suppress damaging overexpression [38,42]. The fact that the up-regulation of X-linked genes in *Drosophila* is male-specific, whereas that in the mouse is not, suggests that the mechanisms by which up-regulation is achieved may be fundamentally different in the two organisms, despite the presence in mammals of homologues of several of the *Drosophila* dosage-compensation complex components [44,45]. Unravelling the up-regulation mechanism in mammals and defining how it interacts, if at all, with *Xist*-mediated silencing to optimise expression of X-linked genes are now questions of particular interest.

## Materials and Methods

### Cells and cDNA preparation.

The mouse ES cell lines PGK12.1 (129 × PGK hybrid) [46], CCE/R (129/sv) [47], and 3F1 (*129/sv* × *castaneous* hybrid) [25] were cultured as previously described [37]. Differentiation was induced by replating on nonadherent plastic dishes in the absence of leukaemia inhibitory factor (LIF). Adult control cells were thymic lymphocytes from 4-wk-old *Balb/c* mice. ICM cells were prepared from cultured *Balb/c* mouse embryos at the early blastocyst stage by the immunosurgery procedure of Solter and Knowles [48], as previously described [49]. Embryos were sexed by testing (by PCR) the trophectodermal material remaining after immunosurgery for presence of DNA encoding the male-specific, Y-linked antigen Sry.

Total RNA was extracted from ES cells using the RNeasy mini kit (Qiagen). For ICM, RNA was extracted with the RNAqueous-Micro kit and amplified with the MessageAmp II aRNA amplification kit (both from Ambion). cDNA was prepared with RT-Superscript-III (Invitrogen), purified with the Qiagen PCR purification kit, and labelled with Cy3 or Cy5 (Amersham) using Invitrogen Bioprime labelling kits (see Text S1 for details).

### cDNA microarrays.

The NIA 15K mouse cDNA library [14,50] was purchased through the UK Medical Research Council and printed in duplicate onto glass slides by the Genomics and Proteomics Laboratory, University of Birmingham (http://www.genomics.bham.ac.uk) using an Advalytix Automated Hybrydization Station. The library contains 15,247 cDNA clones with an average insert size of 1.5 kb. The ES cell data presented here are derived from 252 X-linked clones (corresponding to 180 named genes) and 6,945 autosomal clones (corresponding to 5,085 named genes) that consistently gave above-background signals with ES cell cDNAs. cDNAs from female and male ES cells at the same stage of differentiation were labelled with Cy3 and Cy5, and equal amounts (80∼120 pmol) were co-hybridised to arrays overnight at 42 °C. After labelling, slides were washed and then scanned using a GenePix 4000A scanner. PMT settings were set so as to balance overall signal in the Cy3 and Cy5 channels. Scans were automatically aligned using GenePix (version 6.0) software and then “cherry-picked” manually to eliminate abnormal spots. Microarray data was extracted by Genepix (version 6.0) and normalised by Gepas software. Clustering analysis used the TIGR MultiExperiment Viewer, TMEV [21] (http://www.tigr.org/tdb/tgi). Detailed analytical procedures can be found in Text S1.

### Real-time PCR and SNP analysis.

Expression patterns of four genes (*Maoa1*, *Prps1*, *Ssr4*, and *Smc1l1*) were quantified by real-time PCR using SYBR Green PCR master mix (ABI) and an ABI 7900 Detection System. The primer sequences are listed in Table S4. Allele-specific quantification of *Zfp185* was by radioactive PCR with two forward primers (Table S4), specifically recognising *129* and *castaneous* alleles. *ActB* was used as a control. The PCR reaction comprised 5 μl 2× buffer, 1 μl cDNA, and 2.5 pmol each of primers in a total volume of 10 μl.

SNPs distinguishing *m.m.domesticus* (*129*) and *m.m.castaneous* X-linked genes were identified using Ensemble SNPView. Allele-specific expression was analysed by restriction enzyme digestion following amplification of cDNA from undifferentiated (day 0) 3F1 cells by PCR. Primers, enzymes, and expected products are listed in Table S5 and detailed procedures are given in Text S1.

### RNA FISH.

RNA FISH was carried out as described by Okamoto et al. [17]. Briefly, cells were cytospun to glass slides and fixed in 3.5% paraformaldehyde in PBS for 30 min at room temperature and permeabilised with 0.5% Triton X100 in PBS + 2 mM vanadyl ribonucleoside complex (Biolab) for 10 min on ice. Cells were then dehydrated, hybridised, and counterstained with DAPI. The 6-kb GPT16 *Xist* probe [51] was labelled with Spectrum Green-dUTP (Vysis) by nick translation, according to the manufacturer's protocol.

## Supporting Information

Figure S1 Expression of *Xlr5* and *Xlr3b* during ES Cell Differentiation (A) Microarray-derived expression of the *Xlr5* and *Xlr3b* genes in male (CCE/R) and female (PGK12.1) ES cells, as indicated, at different days of differentiation.
(B) Microarray-derived expression of the *Nanog*, *Pou5f1*, and *Zfp42* genes in female (PGK12.1) ES cells at different days of differentiation.
(C) Microarray-derived expression of the *Xist* gene in female (PGK12.1) ES cells at different days of differentiation. Expression is relative to autosomal genes (log_2_X:A ratio).
(38 KB PPT)

Figure S2 Distribution of Expression Levels of X-Linked and Autosomal Genes in Male and Female ES Cells X-linked gene expression in undifferentiated and differentiated ES cells shows a distribution skewed towards higher expression levels. In undifferentiated cells only, the median expression is higher in females than in males. Autosomal genes show a similar distribution and skewing, but with no distinction between males and females, or change with differentiation; the distributions for genes on Chromosomes 2 and 10 are shown as examples.
(45 KB PPT)

Figure S3 Expression of Genes on Individual Autosomes by Microarray Analysis Expression levels of genes on individual Chromosomes 1–19, relative to expression from all autosomes (n:A ratio), in female (red) and male (blue) ES cells through differentiation are shown. None of the autosomes show any consistent difference between males and females or any change with differentiation, with the exception of Chromosome 11, whose genes are consistently more strongly expressed in males, and Chromosome 12, whose genes are consistently more strongly expressed in females. However, these expression differences are small compared to those shown by X-linked genes.
(60 KB PPT)

Figure S4 Fold Change in Expression of Some X-Linked Genes During Female ES Cell Differentiation (A) Array-derived expression ratios of *Maoa1*, *Prps1*, *Ssr4*, and *Smc1l1* at different days of differentiation in ES cell. Data for each gene was normalised to day 0.
(B) Real-time PCR validation of array-derived expression ratio. *ActB* was used as an endogenous, internal control and the data was normalised to day 0 values thereafter, as in (A).
(34 KB PPT)

Figure S5 Correlation between Genes Inactivated in PGK12.1 and 3F1 ES Cells after 7 d of Differentiation The graph shows how levels of expression of individual X-linked genes change after 7 d of differentiation in two female ES cell lines: PGK12.1 (*x*-axis) and 3F1 (*y*-axis). For each cell line, cDNAs from undifferentiated and differentiated cells were co-hybridised to the same slide, and the difference in expression was calculated as an M value (day 7:day 0 ratio, log_2_ scale). Pearson product moment correlation coefficients (*r* value) and probabilities of chance correlation (*p*) are shown. These experiments were carried out with an NIA15K “half library”, and the number of data points is less than shown in other figures.
(34 KB PPT)

Figure S6 Allelic Expression of Zfp185 during Female ES Cell Differentiation (A) Two forward primers (Table S4) hybridize selectively to the SNP that distinguishes the *129* and *castaneous* alleles of *Zfp185* in 3F1 hybrid ES cells.
(B) Allele-specific analysis shows reduced expression of *Zfp185* from the *129* allele by day 7 of 3F1 differentiation.
(63 KB PPT)

Figure S7 Measured Frequency of “Same-Cluster” Gene Pairs in Groups of Five or More Gene Pairs Separated by Increasing Distances The blue line shows the measured frequency of gene pairs in which both genes were in the same cluster as group size increased to incorporate gene pairs that were increasingly far apart. The 20 closest gene pairs (up to 40 kb apart) are shown in Table S3. The red line shows the frequencies representing the 95th percentile for each group, calculated as described above.
(32 KB PPT)

Table S1 Clones and Corresponding Genes in Cluster 4 Chromosome locations are from the online data provided for the NIA15K array (http://www.nia.nih.gov).
(37 KB DOC)

Table S2 Gene Distribution along the X Chromosome (A) Distribution along the X chromosome of clones in clusters 1–4.
(B) Distribution along the X chromosome of genes in clusters 1–4.
(C) Distribution along the X chromosome of genes in clusters 1–4 as a proportion of genes in each cluster.
(52 KB DOC)

Table S3 Relationship between the Proximity of Genes within a Gene Pair and Their Presence within the Same or Different Clusters For the purposes of statistical analysis, pairs were counted as one if they were within the same cluster and zero if they were in different clusters; see also Figure S7.
(46 KB DOC)

Table S4 Primer Pairs Used for Real Time PCR Validation of Microarray Data (35 KB DOC)

Table S5 Primers and Restriction Enzymes Used for Allele-Specific Analysis of Selected X-Linked Genes (42 KB DOC)

Text S1 Details of Statistical Procedures Used for Analysis of Microarray Data and Technical Procedures Used for cDNA Preparation, PCR, and SNP analysis (46 KB DOC)

### Accession Numbers

The National Center for Biotechnology Information (NCBI) unigene cluster IDs (http://www.ncbi.nlm.nih.gov) for the genes mentioned in the text are as follows: *Acsl4* (Mm.391337), *Brodl* (Mm.100112), *Gm784* (Mm.298000), *Jarid1c* (Mm.142655), *Nanog* (Mm.440503), *Ogt* (Mm.259191), *Pctk1* (Mm.102574), *Pgr151* (Mm.336164), *Phka2* (Mm.350712), *Pou5f1*/*Oct4* (Mm.17031), *Sry* (Mm.377114), *Tsix* (Mm.435573), *Xist* (Mm.435573), *Xlr3b* (Mm.336117), *Xlr5* (Mm.435653), *Xlr5c* (Mm.255790), *Xlr5d* (Mm.435653), *Zfp42*/*Rex1* (Mm.285848), and *Zfp185* (Mm.1161).

## Abbreviations

ES cells: embryonic stem cells
FDR: false discovery rate
FISH: fluorescence in situ hybridisation
ICM: inner cell mass
RTQ-PCR: real-time quantitative PCR
SNP: single nucleotide polymorphism
Xic: X inactivation centre
