# Supplementary material for: Dosage Compensation in the Mouse Balances Up-Regulation and Silencing of X-Linked Genes
Source: PLoS Biol. 2007 Dec 11;5(12):e326. doi: 10.1371/journal.pbio.0050326 (PMC2121114; doi:10.1371/journal.pbio.0050326)
Supplement: Supplementary file 1 [file pbio.0050326.sd001.doc]

**TEXT S1**

**SUPPLEMENTARY METHODS**

*Microarray data analysis*

GenePix software was used to quantify fluorescence intensity for each feature and the local background on the array. Normalisation was then conducted using Gepas software ([www.gepas.org](http://www.gepas.org/)) with global loess approach (Smyth and Speed, Methods 31, 265-271, 2003), which is based on the assumption that the total integrated intensity (after background subtraction) across all spots on one array is equal from both channels and will not be affected by a small number of differentially expressed genes (eg. the X-chromosome genes or other sex-specific genes in our study). The array contains over 15K cDNA sets therefore we can assume overall autosomal gene expression is equal between female and male mouse tissue and ES cells (or embryoid bodies at the same day of differentiation), and should remain unchanged during ES cell development. The M (log-ratios of expression) and A values (log-intensity of expression) of each feature on the array were then obtained and used for further analysis.

For final analyses the data set was filtered so as to include only named, validated genes (ie. unnamed genes designated “NA” or “unknown” in the clone list were eliminated). This gave us 9,582 clones out of the original 15,247. For differentiation timecourse analysis, only clones giving signals on at least 13 of the 22 slides were included, thus eliminating genes whose expression levels were so low as to give unreproducible signals on this array. Final numbers of X-linked clones/genes remaining post-filtering for each type of analysis are given in the text or Figure legends.

To address the X-linked gene inactivation during mouse ES cell differentiation, we

used M values to define red:green (ie. female:male) fluorescence intensity ratios at different times of differentiation (M = log_2_ R - log_2_ G). The M values of the duplicated features on the array were averaged and data were filtered to use only clones that have valid names and have data on at least half of the experiments, including biological repeats and various differentiation time points. The median values were then obtained from the X-linked and autosomal clones. The median M values for autosomal clones are all very close to 1 (0.98~1.01) as expected. The median M values for X-linked clones of biological repeats were averaged to obtain the final M value.

For separate analysis of X:A ratio changes in female and male mouse ES cells during ES cell differentiation, base-2 logarithms for red and green intensities of each spot were calculated from M and A values.

M=log_2_ R - log_2_ G, A=(log_2_ R + log_2_ G)/2

log_2_ R = A + M/2, log_2_ G = A - M/2

Data for the duplicates of each clone were combined and data were filtered as above. The average log_2_R (and log_2_G) values for each clone were then obtained from biological repeats. The median values for the autosomal clones were also calculated and the X:A ratio of each X-linked clone derived.

Cluster analysis of the 252 X-linked clones was carried out by TMEV ([www.tigr.org](http://www.tigr.org/)) using KMC calculated mean with Pearson correlation distance metric. Ontology analysis of gene clusters used FatiGO+ ( [www.fatigoplus.org](http://www.fatigoplus.org/) ).

*Cluster enrichment analysis for the genes present near the X-inactivation centre*

8 of the 40 genes that were present in the region neighboring the X-inactivation centre (*Xic*, 85-108Mb) belonged to the set of 21 genes in cluster 4 (supplementary Table S2). To determine the significance of this, we constructed a 2x2 contingency table (shown below) and used Fisher’s Exact Test to show that the *Xic* region was significantly enriched in cluster 4 genes as compared to genes from other clusters (*P=* 0.0378). The same test showed that none of the other X chromosome regions listed in Table S2 were significantly enriched in genes from any one of the four clusters.

|  | In cluster 4 | Not in cluster 4 |
| --- | --- | --- |
| Xic bin (85-108Mb) | 8 | 32 |
| Outside the Xic bin | 13 | 152 |

*Testing the relationship between gene proximity and time of silencing*

To test the hypothesis that genes that are in close proximity to each other are silenced at the same times during ES cell differentiation (ie. are present in the same cluster), we took all the genes that had been assigned to a cluster and for which positional information was available (118, Mouse Genome Informatics (MGI) [www.informatics.jax.org](http://www.informatics.jax.org/)) and listed the possible gene pairs in order of the distance between them. We then distinguished those pairs whose genes were in the same cluster (same-cluster gene pairs) or in different clusters. Remarkably, the five closest gene pairs (0-2.8Kb apart) were all in the same cluster, as were 12 of the first 20 pairs. These are listed in Table S3. To estimate the significance of this, we wrote a programme (in R) that randomly shuffled the gene-pair list (1000 iterations) and calculated the frequency distribution of same-cluster gene pairs in groups comprising the closest *n* gene pairs (where n≥ 5). Where the observed frequency fell at or above the 95^th^ percentile of this calculated frequency distribution, it was considered significantly enriched in same-cluster pairs. As shown in Figure S7, the observed frequency of same-cluster gene pairs was frequently at or above the 95^th^ percentile for the first 20 gene pairs (ie. n = 5-20, with genes up to 40Kb apart), but declined thereafter, gradually approaching the median value.

*cDNA preparation*

Total RNA was extracted using the RNeasy kit (Qiagen), according to the manufacturer’s protocol and treated with DNaseI (Qiagen). First strand cDNAs were reverse transcribed from 3μg total RNA in a reaction containing 4μl 5x first-strand buffer, 1 μl 0.1M DTT, 1μl oligo dT primer (50 μM), 200unit RT-Superscript III transcriptase (RT-SSIII, Invitrogen), 40unit RNAase inhibitor (Boehringer Mannheim) made up to 20μl. The cDNA was purified with a Qiagen PCR purification kit. 200μg aliquots of cDNA were labelled with Cy3 or Cy5 fluorophore (Amersham) using Invitrogen Bioprime random primer labelling kits. Briefly, the cDNA was annealed with 20μl 2.5x random primers at 95°C for 2 minutes. 1.2mM each of dATP, dGTP, dTTP and 0.6mMdCTP, 1ul of Cy dye and 1ul of Klenow fragment were then added to the reaction and incubated overnight at 37°C. The labelled samples were purified again and equal amounts (80~120pmol each) of Cy3- and Cy5-labelled cDNAs were combined and hybridised to arrays overnight at 42°C.

*PCR and SNP analysis*

Microarray-determined expression levels of selected genes were validated by real time PCR using the primers listed in Table S4. Allele specific expression was analysed by restriction enzyme digestion following amplification of cDNA from undifferentiated (Day 0) 3F1 cells by PCR. Primers, enzymes and expected products are listed in supplementary Table S5 The amplification was carried out in 30μl of 1.1x ABgene PCR master mix using gene specific primers (0.5 μl of each primer of 100pmol/μl per reaction) in the presence of α^32^ P (1μCi per reaction). The PCR products were extracted using Qiagen gel extraction kit followed by restriction enzyme digestion. The digested products were separated on a 7.5% polyacrylamide gel that was then vacuum dried and scanned using Typhoon^TM^ 9200 PhosphorImager (Amersham Biosciences). The scanned images were quantified using the ImageQuant^TM^ TL v2003.01 (Amersham Biosciences).The timing of allele specific inactivation was determined in a similar way except the PCR was carried out without ^32^P. The digested products were separated on a 7.5% polyacrylamide gel and then exposed to ethidium bromide (25μl per 100ml of 1X TBE buffer) in 1X TBE buffer.
