# Supplementary material for: Dosage Compensation in the Mouse Balances Up-Regulation and Silencing of X-Linked Genes
Source: PLoS Biol. 2007 Dec 11;5(12):e326. doi: 10.1371/journal.pbio.0050326 (PMC2121114; doi:10.1371/journal.pbio.0050326)
Supplement: Supplementary file 2 [file pbio.0050326.st001.doc]

**SUPPLEMENTARY TABLES**

**Table S1**

| Clone ID | Gene Name | Position on Chr. X (Mb) |
| --- | --- | --- |
| H3141g04  H3130f05  H3112f08  H3112f10  H3011f03  H3112f09  H3130h12  H3112f11  H3006b03  H3094h09  H3148f07  H3127d03  H3005f06  H3020h07  H3156d08  H3044c12  H3151f03  H3057h06  H3023b04  H3026e10  H3150g01  H3097d09  H3120d05  H3081a12  H3146a06  H3159e11 | *Suv39h1*  *Crsp2*  *Rpl3*  *Rpl3*  *Rpl3*  *Rpl3*  *Rpl3*  *Rpl3*  *Il13ra1*  *Bcorl1*  *5830467J12Rik,6720401G13Rik*  *Bgn*  *Irak1*  *Gdi1*  *2810002O09Rik*  *Msn*  ***Xist***  *Slc16a2*  *2610529C04Rik*  *Gm784*  *Itm2a*  *Sh3bgrl*  *Armcx3*  *Acsl4*  ***Jarid1c***  *BC022960* | 6.35  11.01  11.53  11.53  11.53  11.53  11.53  11.53  28.66  40.72  42.90  65.04  65.57  65.86  86.83  87.50  94.83  95.10  97.37  97.82  98.80  100.50  124.76  132.21  142.12  159.48 |
