# Supplementary material for: Dosage Compensation in the Mouse Balances Up-Regulation and Silencing of X-Linked Genes
Source: PLoS Biol. 2007 Dec 11;5(12):e326. doi: 10.1371/journal.pbio.0050326 (PMC2121114; doi:10.1371/journal.pbio.0050326)
Supplement: Supplementary file 3 [file pbio.0050326.st002.doc]

**SUPPLEMENTARY TABLES**

**Table S2**

**A.**

| *Mb* | *Cluster 1* | *Cluster 2* | *Cluster 3* | ***Cluster 4*** | *Total* |
| --- | --- | --- | --- | --- | --- |
| 0 - 14  15 - 30  31 - 54  55 - 84  85 - 108  109 - 140  141 - 160 | 11  7  8  7  14  6  7 | 7  6  11  15  14  14  19 | 7  14  4  16  14  8  14 | 8  1  2  3  8  2  2 | 33  28  25  41  50  30  42 |
| Total | 60 | 86 | 77 | 26 | 249 |

**B.**

| *Mb* | *Cluster 1* | *Cluster 2* | *Cluster 3* | ***Cluster 4*** | *Total* |
| --- | --- | --- | --- | --- | --- |
| 0 - 14  15 - 30  31 - 54  55 - 84  85 - 108  109 - 140  141 - 160 | 7  6  7  7  8  4  7 | 7  6  8  15  13  12  13 | 6  11  4  14  11  7  11 | 3  1  2  3  8  2  2 | 23  24  21  39  40  25  33 |
| Total | 46 | 74 | 64 | 21 | 205 |

**C.**

| *Mb* | *Cluster 1* | *Cluster 2* | *Cluster 3* | ***Cluster 4*** |
| --- | --- | --- | --- | --- |
| **0 - 14**  **15 - 30**  **31- 54**  **55 - 84**  **85 - 108**  **109 - 140**  **141 - 160** | 0.15  0.13  0.15  0.15  0.17  0.09  0.15 | 0.09  0.08  0.11  0.20  0.18  0.16  0.18 | 0.09  0.17  0.06  0.22  0.17  0.11  0.17 | 0.14  0.05  0.10  0.14  0.38  0.10  0.10 |
