# Supplementary material for: Dosage Compensation in the Mouse Balances Up-Regulation and Silencing of X-Linked Genes
Source: PLoS Biol. 2007 Dec 11;5(12):e326. doi: 10.1371/journal.pbio.0050326 (PMC2121114; doi:10.1371/journal.pbio.0050326)
Supplement: Supplementary file 4 [file pbio.0050326.st003.doc]

**SUPPLEMENTARY TABLES**

**Table S3**

| Gene A | Gene B | Distance (Kb) | Counter | Cluster |
| --- | --- | --- | --- | --- |
| *Ctps2* | *S100g* | 0 | 1 | 2 |
| *Idh3g* | *Ssr4* | 0.13 | 1 | 3 |
| *Hnrph2* | *Gla* | 0.19 | 1 | 2 |
| *Rpl36a* | *Gla* | 2.00 | 1 | 2 |
| *Renbp* | *Hcfc1* | 2.78 | 1 | 3 |
| *F8a* | *Vbp1* | 2.99 | 0 |  |
| *Slc6a8* | *Bcap31* | 3.91 | 0 |  |
| *Suv39h1* | *Was* | 5.32 | 0 |  |
| *Flna* | *Emd* | 8.14 | 0 |  |
| *Tmem32* | *Slc9a6* | 11.5 | 0 |  |
| *Rpl36a* | *Hnrph2* | 12.4 | 1 | 2 |
| *Taz* | *Gdi1* | 15.5 | 1 | 1 |
| *Tktl1* | *Flna* | 18.2 | 1 | 3 |
| *Rbm3* | *Wdr13* | 19.1 | 0 |  |
| *Taz* | *Emd* | 29.0 | 1 | 1 |
| *Fmr1nb* | *Fmr1* | 30.2 | 1 | 2 |
| *Rbm10* | *Pctk1* | 31.7 | 1 | 3 |
| *Slc6a8* | *Dusp9* | 36.6 | 1 | 3 |
| *Hprt1* | *Phf6* | 38.2 | 0 |  |
| *Irak1* | *Hcfc1* | 39.1 | 0 |  |
