# Supplementary material for: Dosage Compensation in the Mouse Balances Up-Regulation and Silencing of X-Linked Genes
Source: PLoS Biol. 2007 Dec 11;5(12):e326. doi: 10.1371/journal.pbio.0050326 (PMC2121114; doi:10.1371/journal.pbio.0050326)
Supplement: Supplementary file 5 [file pbio.0050326.st004.doc]

**SUPPLEMENTARY TABLES**

**Table S4**

| Gene | Forward | Reverse |
| --- | --- | --- |
| *Maoa* | tgttggtggagcttatgtgg | caccacggaatgggtaagtt |
| *Prps1* | ccaccaggacttatcccaga | gccaatttccacacaggtct |
| *Ssr4* | gcaagaacagggtccagaac | cctgatatcgacccacatcc |
| *Smc1l1* | acaagaagcggatggacaag | ttccgagtccttttccttga |
| *ActB* | cgtgaaaagatgacccagatca | tggtacgaccagaggcatacag |
| *Zfp185* | ctgcatggtcactgttatcg (*Cast.*) | ccctttgatagttgagctgga |
|  | ctgcatggtcactgttatca (*129*) |  |
