# Supplementary material for: Dosage Compensation in the Mouse Balances Up-Regulation and Silencing of X-Linked Genes
Source: PLoS Biol. 2007 Dec 11;5(12):e326. doi: 10.1371/journal.pbio.0050326 (PMC2121114; doi:10.1371/journal.pbio.0050326)
Supplement: Supplementary file 6 [file pbio.0050326.st005.doc]

**SUPPLEMENTARY TABLES**

**Table S5**

| Gene | Forward | Reverse | Enzyme | Restriction pattern (bp) | |
| --- | --- | --- | --- | --- | --- |
|  |  |  |  | *129* | *Cast.* |
| *Acsl4* | gcacaacagaagggggtaga | tttcagcttgaaggcatctg | TaqI | 82, 116 | 82, 54, 62 |
| *Brodl* | ggatccaatttcaggcaaa | tctggaatcggctccatatc | Tsp509I | 288 | 171, 117 |
| *GM784* | accagagcctccacagctta | ccttccccagcttcattaca | MaeII | 112, 131 | 243 |
| *Jarid1c* | cctggcagcagctgtacata | tagcccaggtgtcaaaggac | NspI | 154, 187 | 341 |
| *Phka2* | gccacctctctcttcctcct | ttcatcaatggcctcaagtg | MaeII | 234 | 147, 87 |
| *Pgr15l* | agggccaacaggatcagtc | agaaaacgagccagagcaaa | DpnII | 243 | 183, 60 |
| *Ogt* | tctcgagttgcagcttctca | catgtggtcaggtttgttgc | AvaI | 210, 38 | 248 |
| *Pctk1* | ccaggtatcctgtccaatgagg | aggccaatggttgggttgac | PvuII | 540 | 250, 290 |
